# Item Selection and Content Validity of the Risk Factors of Post-Intubation Tracheal Stenosis Observation Questionnaire for ICU-Admitted Patients

**Published:** 2017

**Authors:** Roya Farzanegan, Behrooz Farzanegan, Maryam Alehashem, Mahdi Zangi, Sharareh R. Niakan Kalhori, Kambiz Sheikhy, Habib Emami, Mohammad Behgam Shadmehr

**Affiliations:** 1 Tracheal Diseases Research Center (TDRC), National Research Institute of Tuberculosis and Lung disease (NRITLD), Shahid Beheshti University of Medical Sciences, Tehran, Iran.; 2 Department of Health Information Management, School of Allied Medical Sciences, Tehran University of Medical Sciences, Tehran, Iran.; 3 Tobacco Prevention and Control Research Center(TPCRC), NRITLD, Shahid Beheshti University of Medical Sciences, Tehran, Iran

**Keywords:** Tracheal stenosis, Intubation, Risk factor, Questionnaire, Content validity

## Abstract

**Background:**

Laryngotracheal stenosis as a late complication of prolonged endotracheal intubation is a life-threatening event. In order to determine the related risk factors for this complication, which may vary among different countries, designing a valid questionnaire is necessary. The aim of this study was to select the items and evaluate the face and content validities of a questionnaire developed for assessment of risk factors of post-intubation tracheal stenosis (PITS) in patients admitted in the intensive care unit.

**Materials and Methods:**

A mixed method study design was used in four steps in 2015, i.e., 1) a literature review, 2) focus groups with five experts in the field, 3) consultations with intensive care unit (ICU) specialists and thoracic surgeons, and 4) evaluation of content and face validity with 15 experts in a scientific panel using two self-administered questionnaires. Content validity index (CVI) was computed for individual items as well as the overall scale.

**Results:**

We extracted the items from different sources of information. An initial version of the 52-item questionnaire was developed and classified into four domains including patient characteristics, intubation features, equipment-drugs, and complications. The items with an excellent modified kappa were included in the questionnaire. Five questions received more criticism instead of support and were removed (Item-CVI<0.55, fair modified kappa). The ones with an Item-CVI > 0.60 and a good modified kappa were revised, merged, or retained. The new 43-item questionnaire found a scale-level CVI, averaging (Scale-CVI/Ave) of 0.91.

**Conclusion:**

The PITS risk factors questionnaire was developed and validated through item selection, expert opinions, and content validity index.

## INTRODUCTION

Endotracheal intubation is performed in patients who require mechanical ventilation. Tracheal stenosis, one of the worst complications of prolonged intubation, is rare but life-threatening with remarkable morbidities (1). Although tracheal stenosis can occur following other etiologies such as trauma, inflammatory disease, burns, tumors, or idiopathic reasons (2), post-intubation tracheal stenosis (PITS) is the most common etiology for reconstructive airway surgeries (3).

During the period of intubation, the direct pressure of the cuff and/or the tip of the tube on the mucosa and the subsequent ischemia stimulate an inflammatory reaction, leading to mucosal edema, granulation tissue formation, fibrosis, cartilage destruction, and finally, tracheal stenosis after extubation. Another mechanism could be direct trauma subsequent to a forceful intubation of critically injured patients by less experienced medical staff.

The incidence rate of PITS varies from country to country due to various reasons, like the varying prevalence of the etiological factors, expertise of pre-hospital emergency medical staff, number of ICUs, the experience of ICU staff with non-traumatic intubation, and the quality of the equipment. It was estimated to be 4.6% in the United Kingdom, and 20% in India (1, 3). In a prospective study by Stauffer et al., it was reported to be 19 % (4).

There are numerous potential risk factors contributing to PITS, such as cuff pressure, size of the tube in proportion to the tracheal lumen, irritation from cuff materials, age, sex, and bacterial infection (5, 6).

Other risk factors may include improper placement of the endotracheal tube, long duration of intubation, the severity of respiratory failure, and insufficient training of the ICU staff for handling endotracheal tubes (7, 8). Volpi et al. indicated that some underlying diseases like diabetes mellitus, congestive heart failure, stroke, and tuberculosis can enhance the probability of laryngeal injury (9).

Although most researchers consider cuff pressure to be the main cause of tracheal stenosis (10), it may occur despite using tubes with high volume-low pressure cuffs (10).

The literature shows that 10% of the patients with PITS may remain undiagnosed for over 10 years, or even be wrongly treated for asthma (11). In a patient with a history of intubation for over 24 hours and clinical manifestations of airway obstruction (dyspnea, cough, stridor, wheezing), PITS should be considered as a differential diagnosis (12, 13). The natural history of tracheal stenosis can be modified by early diagnosis (1). To best of our knowledge, there is no screening program for those patients who are discharged from ICUs after prolonged intubation. Rigid bronchoscopy and dilatation of the stenosis is the best initial management and could be repeated several times as required. However, the frequent hospital admissions and general anesthesia lead to a significant physical, psychological, and economic surcharge on both, the patients and the health system. Ultimately, in most cases, tracheal resection and reconstruction would be required for optimal treatment (14).

In Iran, traffic accidents constitute one of the most fatal injuries. These occur commonly among the young population (15). Our database for all patients with tracheal diseases (Alborz database), which includes more than 2300 patients in the previous two decades (16–21), shows that traffic accidents are the main cause of hospitalization and intubation in most of our patients.

Previous study estimated a 65% increase in traffic injuries in developing countries in the next 20 years (22). Therefore, it can be inferred that the incidence rate of PITS would become increasingly remarkable in developing countries in the upcoming years. Consequently, the health policies should also focus on preventive methods like screening programs. To accomplish this goal, longitudinal epidemiological studies should be conducted to determine the risk factors of PITS and design a practical screening program. To best of our knowledge, in spite of many studies to determine the risk factors of PITS (23–27), there is no valid questionnaire to fulfill the goal of prevention measurement.

Several studies have emphasized on the content validity of an instrument to decrease the measurement errors, and improve the efficacy and appropriateness of the captured data (28, 29). In the current study, we designed a reliable questionnaire and then validated it through the content validity index (CVI). This index measures the adequacy of the selected items to represent the content that the questionnaire is supposed to measure (29, 30). The index was applied to estimate the risk factors of PITS in adult patients with a history of prolonged endotracheal intubation.

The aim of this study was to select the items and evaluate the face and content validities of a questionnaire developed for assessment of risk factors of post-intubation tracheal stenosis (PITS) in patients admitted in the intensive care unit.

## MATERIALS AND METHODS

### Study design

A mixed method study design was chosen to develop a questionnaire for assessment of the risk factors of PITS at the Tracheal Diseases Research Center in 2015.

### Literature review

A review of the literature was performed to find any validated instrument for tracheal stenosis. PubMed, Google Scholar, Scientific Information Database (SID), and Cochrane databases were searched without limiting the dates of publication by two experts during 2012 and updated in 2015. Articles in the English and Persian languages were selected. The keywords used to identify the reported risk factors for PITS were “tracheal stenosis/stricture/lesion”, “laryngotracheal stenosis/stricture/lesion”, “risk factors, “, “epidemiology” “predisposing factors”, “intubation,” and “airway”. The reference lists in the relevant articles were also used. We did not find any validated questionnaire regarding the risk factors of PITS in the literature. Therefore, the review authors extracted all the related risk factors studied or mentioned in the articles. Some of those risk factors are shown in Table 1. Then, the main authors checked the shortlisted risk factors and designed a preliminary questionnaire based on their work. The questionnaire was subsequently revised after considering the experts’ opinions.

**Table 1.** The risk factors associated with early or late complications after endotracheal intubation

**Authors**	**Journal**	**Year**	**Place**	**Method**	**Studied or mentioned Risk factors**
Fishman et al (55)	The annals of thoracic surgery	1969	-	-	Inadequate patient care
Miller and Sethi (56)	Annals of surgery	1969	Kansas	Case series/9 cases	Prolonged cuff intubation
Andrews and Pearson (57)	Annals of surgery	1971	Canada	Prospective Cohort/tracheostomy/121 cases.	Type of cuff, size of cuff, hypotension, duration of intubation, steroids,
Balluch (58)	HNO	1983	German	-	the deficit of fibrin-stabilizing factor XIII
Kastanos (59)	Critical Care Medicine	1983	Spain	Prospective/19 cases	Severe respiratory failure, high cuff pressure, and secretion infection
Whited (60)	Laryngoscope	1984	Cincinnati	Prospective/200 cases	Period of intubation
Bishop(61)	Chest	1989	Seatle	Commentary	Tube size, cuff pressure, prolonged intubation, tube movement,
Grillo and Donahue (62)	Seminars in thoracic and cardiovascular surgery	1996	USA	Review	Cuff, stoma
Vila et al. (63)	European Archives of Oto-Rhino-Laryngology	1997	Spain	Prospective-endoscopic study/39 cases	Period of intubation
Beebe (64)	Seminars in Anesthesia, Perioperative Medicine, and Pain	2001	Minnesota	review	Prolonged intubation/cuff pressure/tracheal tube
Yamada et al. (65)	Arch Dis Child Fetal Neonatal ED	2001	Japan	Case report/2 cases	Local infection
Hocking et al. (66)	Anaesthesia	2001	UK	Experimental study/52 females	Airway obstruction with cricoid pressure and lateral tilt.
Koufman et al. (67)	Otolaryngology - Head and Neck Surgery	2002	New York	Review	Aspirated gastric juice, reflux
Zagalo et al. (68)	Surgical and radiologic anatomy : SRA	2002	Portugal	Case series/20 cases	Duration of intubation, age
Papla, et al. (10)	Pol J Pathol	2003	Kraków	Cross section/42 cases	Chemical agents, local infection, hypotension
Mol et al. (8)	South African Journal of Surgery	2005	Bloemfontein	Questionnaire survey/112 cases	ICU care/Cuff pressure/adequate information of ICU staff
Esteller-Moré et al. (23)	European Archives of Oto-Rhino-Laryngology	2005	Spain	Prospective study/605 cases	APACHE II, age, sex, ICU stay, hospital stay, duration of intubation, cause of intubation, poor status condition, local infection
Atlas (43)	Journal of Clinical Monitoring and Computing	2005	New Jerssy	Experimental/a math model	Cuff pressure & body temperature
Rangachari et al. (69)	Indian J Crit Care Med	2006	India	Prospective study/51 cases	Emergency intubation, duration of intubation, tube size
Griesdale et al. (24)	Intensive care medicine	2008	Canada	Prospective study/136 cases	Level of intubator, difficult intubation, medications, techniques
Sole et al. (42)	American journal of critical care	2009	Florida	Pilot study/10 cases	Cuff pressure & positioning on bed, endotracheal tube suction, lack of patient-ventilator coordination and cough
Young and Doyle (70)	Current Respiratory Medicine Reviews	2012	UK/USA	Review	Preventing Ventilator-Associated Pneumonia - The Role of the Endotracheal Tube
Herrak et al. (71)	Egypt. J. Chest Dis. Tuberc	2013	France	Retrospective//174 cases	Tip of rigid intubation tube, cuff pressure
Hong (72)	Korean J Crit Care Med	2014	Korea	Editorial	Shape of cuff
Adiguzel et al. (73)	Turkish Thorac. Journal	2014	Istanbul	Cross sectional/2 groups	length of Icu stay; twenty-four-hour intensivist
Lizy et al (41)	Am J Crit Care	2014	Belgium	Prospective-interventional study	Changes in body position in critically ill patients
Memela and Gopalan (74)	Southern African Journal of Critical Care	2014	South Africa	Prospective observational study/35 cases	Intermittent monitoring cuff pressure
Bauchmuller and Faulds (75)	Surgery (Oxford)	2015	UK	Review	Mechanical ventilation
Elmer et al. (76)	Critical care	2015	UK	Secondary analysis of a prospective registry/151 cases	Number of attempts, need for adjuncts to direct laryngoscopy, best Cormack-Lehane grade and training level of final intubator
Alzahrani et al. (77)	BMC anesthesiology	2015	Kingdom of Saudi Arabia	Prospective cohort	Cuff pressure

### Qualitative consultations with ICU specialists and thoracic surgeons

Three focused groups discussions, each with a thoracic surgeon (a professor of surgery with more than 20 years of experience in this field), an ICU specialist (an assistant professor of critical and intensive care, with 8 years of experience), two physicians, and an ICU nurse (with about 8 years of experience in the research field), were conducted. The sessions were in Persian and took about 180 minutes. In the focused groups, any item was discussed and the ideas were shared. Then, the questions were re-designed in four domains including patient characteristics, intubation features, equipment-drugs, and complications.

In order to exclude any difficulty or ambiguity, intensivists who had been working in intensive care units for at least three years and thoracic surgeons involved in the management of tracheal stenosis for more than 10 years were invited to participate in a scientific committee. All the selected questions were expressed and discussed by the main investigators. For the face validity, the experts reassessed each question to eliminate any probable obstacles regarding the physicians’ feedback. The questionnaire including 52 questions was developed and then confirmed in four domains.

### Questionnaire guide

A questionnaire guide was provided for better clarification and comprehensibility of the questions as well as for defining the variables for the respondents. It was uploaded to the website of the research center.

### Evaluation of content validity:

We chose the content validity index (CVI) to ensure adequate ability of the items in each thematic domain to precisely measure the PITS risk factors.

A panel of experts comprising of 10 intensivists (in 10 different ICUs) and five thoracic surgeons (in a tertiary hospital that cares for patients with tracheal stenosis) was approached to evaluate the content and clinical relevance of the first version of the PITS risk factors questionnaire. They were asked to complete a self-administered content questionnaire, which was designed based on a Likert-type scale, in order to prevent recording of any neutral and unspecified answer (29, 31). The CVI of the questionnaire was calculated by a four-point Likert-type scale (consisting of the following options: quite relevant, relevant, approximately relevant, and irrelevant). Moreover, Item-CVI (I-CVI) and Scale-CVI/Average(S-CVI/Ave) were computed (30). The I-CVI information was used to guide us in discarding or revising the items.

In the first round, the panelists received the questionnaire through e-mail or in person. They were asked to return the completed questionnaires to the Research Center within two weeks. After calculating the CVI, some questions were revised or deleted. The comments of the experts in the panel were also considered to develop the new items. Then in the second round, a panel of five experts, who were selected from the same pool of panelists as the first round, evaluated the relevance of the revised items and the S-CVI/Ave was recomputed.

For quantitative evaluation of the face validity, they were asked to complete a self-administered content questionnaire, which was based on a five-point Likert-type scale (comprising of the following options: quite important, important, moderately important, a bit important, and unimportant). Then, the impact score of each item was computed based on its importance. The qualitative method was also applied for face validity. In addition, we asked the panelists to evaluate each question for ambiguity and difficulty.

### Ethical consideration:

The ethics committee of the National Research Institute of Tuberculosis and Lung Diseases (NRITLD) approved the current study.

### Statistical analysis:

To analyze the data collected from focused group sessions, the six-step technique of “theme analysis” was applied. MaxQDA software version 10 (VERBI Software, Marburg, Germany) was used after documenting and transcribing the sessions. In this method, after repeated review and search for meanings and categories, the data were coded. Then, the codes were considered for main and sub-theme review and re-analysis. Thereafter, we defined and named the themes and prepared a map of the themes, followed by final analysis and report writing.

The sum of the number of experts who rated “relevant”and “quite relevant “for each item divided by the number of experts was indicated as I-CVI. The desirable values were a minimum of 0.78 for more than 6 experts regarding Lynn’s criteria (29, 31). In the first round, the items with an I-CVI approximately 0.78 were revised, and those with very low values were discarded. The values of I-CVI were compared with the standards of modified kappa or Kappa-like index (K), which were adjusted for chance agreement on relevance (Fair= K of 4.40–0.59; Good=k of 0.60–0.74; and Excellent= k>0.74)(32). Whenever the number of experts exceeded 10, it was not found necessary to compute the Kappa like-index (29).

In the second round, the S-CVI/Ave was used for consensus estimates of the scale. CVI higher than 0.9 was considered satisfactory for S-CVI/Ave as the average of I-CVIs (33).

To carry out the quantitative evaluation of face validity, the impact score of each item was computed based on the frequency (%) × importance. Scores higher than 1.5 were considered appropriate (34).

Chronbach’s alpha coefficient was used for assessing the internal consistency of each domain of the questionnaire. An alpha value equal to or greater than 0.70 was considered acceptable (34).

## RESULTS

### Qualitative consultations

We developed the initial version of the questionnaire according to the results of the focus group discussions, although it was also evaluated and reconfirmed in the scientific committee. The 52 items obtained from the focus groups findings were categorized into four domains including patients characteristics (11 questions), intubation features (22 questions), equipment-drugs (10 questions), and complications (9 questions). Each domain was designed to measure one group of risk factors.

### Content validity

Fifteen clinicians in the panel were thoracic surgeons and intensivists with vast experience in the field of tracheal stenosis. The mean age and work experience of the panelists were 40.7 and 9.6 years, respectively. Their academic designations were associated professor [2], assistant professor [8], and professor [2].

During the first round, the S-CVI/Ave of the questionnaire was 0.80 with a range of 0.33–1.00. The items with an I-CVI<0.78 were in different domains, even though a majority of them belonged to the intubation domain. Table 2 illustrates the value of I-CVI for each question. The items with an excellent K were included in the questionnaire. Five questions received more criticism instead of support and were removed (I-CVI<0.55, fair K). The ones with I-CVI > 0.60 and good K were revised, merged, or retained. The thoracic surgeons, unlike the intensivists, opined that the items in the intubation domain were somehow relevant to the study objectives. The items numbered 5, 29, 30, 32, and 52 were considered to be candidates for deletions due to a very low agreement among the panelists, CVI<0.55, and a fair K (the removed marked items in the Table 2) as follow:

- In the patient domain: temperature
- In the intubation domain: cricoid pressure, ETCO_2_ detector, type of intubation
- In the complication domain: bedsore

**Table 2.** The values of I-CVI of each item in the questionnaire

**Domains**	**Item number**	**I-CVI**	**Experts’ decision**	**Domains**	**Item number**	**I-CVI**	**Experts’ decision**
	1	0.8667			28	0.5333	Removed
	2	0.6429			29	0.3333	Removed
	3	1.0000			30	0.7333	
	4	0.7333			31	0.5333	Removed
	5	0.6000	Removed		32	0.9333	
**patient**	6	0.6667	Transferred		33	0.7333	
	7	0.6667	Transferred		34	1.0000	
	8	0.7333	Transferred		35	0.7333	
	9	0.8571		**Equipment& Drugs**	36	0.9333	
	10	0.8571			37	1.0000	
	11	0.8667			38	1.0000	
	12	0.93333			39	1.0000	
	13	0.8571			40	0.9333	
	14	0.8667	Merged		41	0.8571	
	15	1.0000			42	0.7333	
	16	0.8000			43	0.7333	
	17	0.8667			44	0.8667	
	18	0.8667			45	0.8667	
	19	0.8667		**Complications**	46	0.8000	
**Intubation**	20	0.8000			47	0.9333	
	21	0.6667			48	0.8571	
	22	0.8000			49	0.9333	
	23	0.7143			50	0.9286	
	24	0.7857			51	0.9286	
	25	0.7333			52	0.4000	Removed
	26	0.6667			S-CVI-first round	0.8001	
	27	0.7143			S-CVI –second round	0.91	

In the patient domain, the items numbered 6, 7, and 8 (i.e., weight, height, and past drug history) were transferred to the other form related to the demographic information of the patients. Item number 14 (type of head injury) was merged with item number 10 (reason of intubation). The experts decided to revise or retain the items that obtained a CVI between 0.60 and 0.74 with a good kappa-like index. Moreover, based on the feedback of the panelists, the sentence structures of items 10 and 11 were also rearranged and changed to the “ICU stay” and the “hospital stay.” After reviewing and scaling the revised questionnaire by the panelists, S-CVI/Ave of the new 43-item questionnaire improved to 0.91 in the second round.

Regarding the face validity, the average impact score was 4.00 (range: 2.87–5.00). Concerning the reliability, Table 3 shows the computed internal consistency of each domain on the questionnaire (Chronbach’s alpha). The final version was a structured and closed-ended questionnaire .

**Table 3.** Chronbach’s alpha in 4 domains

**Domain**	**Chronbach’s alpha**
Patients	0.80
Intubation	0.94
Equipment & Drugs	0.62
Complications	0.89
Total Mean	0.91

## DISCUSSION

Despite several methods for computing the content validity (35), we focused on the consensus estimates. Therefore, the content validity index was used to provide a high-quality measurement of the risk factors of PITS in the patients admitted in the ICU. The CVI was selected for its understandability and ease of calculation and communication (29), and measured the proportion of items that the panelists rated as relevant. To obtain the best results, we worked hard to select and improve the items that were representative of the underlying concept as well as to provide clear instructions for the rating task. Moreover, a strong panel of content experts was carefully chosen to prohibit the bias of the experts. These experts were familiar with the conceptual baseline of the instrument (36). Despite review of the literature by two experts and several focus group sessions, the value of some items was very low, which may be due to the participation of the experts with different specialties in the panel. Most items with a low CVI had been categorized into the intubation domain. There was a disagreement between the thoracic surgeons and the intensivists about rating these questions as “relevance.” Although the intensivists had consensus over most intubation factors, the thoracic surgeons believed more in prolonged intubation rather than the other factors. In addition, our panelists might not have paid adequate attention to the instructions for evaluation of the relevant items. Hence, in the second round, we chose a small group of experts from the same pool of panelists as the first round (29). Thus, based on their information from the former round, they could rate the relevance better. In the second set, the computed SCVI/Ave was higher than 0.9. According to the recommendations of Waltz et al. (37), the computed SCVI/Ave showed a high degree of congruency between the raters on the scale. It meant that the instrument could appropriately measure what it was intended to. In this session, we did not use S-CVI/Universe because the number of experts in the panel was large, which could have led to unacceptable results. On the other hand, a chance agreement was not considered in this method (30).

As we conducted a national study aimed at assessing the risk factors of PITS, our experts decided to use the revised questionnaire without deleting the questions with an S-CVI/Ave of 0.80. The findings of our national study will judge the different opinions of our panelists, regarding the items with very low content validity. The CVI could yield item-level information that provided the extent to which there was an inter-examiner agreement about the relevance of each item to the aim of the questionnaire. This information enabled the researchers to revise or delete the items. However, this method did not involve adjustment for chance agreement on relevance. This limitation was improved by the large number of the experts in the panel (more than 10), which resulted in reduction of the probability of chance agreement (29). Besides, in order to adjust the chance agreement for I-CVIs, a new Kappa-like index was used regarding Fleiss and Cicchetti standards (32,38). More than 10 experts in the panel allowed us to compare the values of I-CVI with the standards of Kappa-like index for modifying the chance agreement that the I-CVI for most of the items was in the excellent range (more than 0.74) (29).

The items that should be removed in order to obtain a valid questionnaire but were kept by our experts for the national study are discussed below.

### Temperature

Factors like head and body movements, duration of intubation, suction, cough, and temperature could change the pressure of the endotracheal tubes (39–42). Some studies have shown the positive relationship between the level of core body temperature and changes in tube cuff pressure (43–44). Therefore, body temperature could be considered as a risk factor for PITS.

### Type of intubation

Endotracheal intubation can be performed through the oral or nasal path. Orotracheal intubation is easier, faster, and less painful than nasotracheal intubation (45). Moreover, in a patient, one larger size tube is used in oral intubation when compared to nasal intubation. In this method of airway management, endotracheal tube is also kinked less than the nasal tube. These two benefits of the orotracheal tube can lead to less airflow resistance. Consequently, the weaning period might be reduced (45). As laryngeal complications are seen more with orotracheal intubation, nasal intubation is preferred for prolonged intubation despite some limitations such as sinusitis and local abscesses (46).

### Bedsore

Bedsores can develop in patients under critical care due to the use of devices, vasoactive medications, and hemodynamic instability (47). The incidence of these sores ranges from 10% to 41% (48–49). The pressure sores might result in serious infection that could increase the ICU stay and prolonged intubation. Therefore, it might indirectly be a risk factor for PITS. In addition, the treatment includes cleaning and dressing the sores as well as changing the patients’ position. During the care of the sores, movement of the patient’s head and body might increase the cuff pressure of the endotracheal tube as well as the pressure on the tip of the tube, which can result in ischemia, inflammation, necrosis, and consequently tracheal stenosis (50).

### ETCO_2_ detector

Malpositioning of the endotracheal tube is a major complication of endotracheal intubation. Timely detection of malposition endotracheal tube plays a vital role in emergent and elective intubation. It could result in increasing the number of intubation attempts, besides hypoxia in the tissues, which may consequently traumatize the trachea.

There are several ways to evaluate the location of the endotracheal tube, like physical examination methods and some techniques such as pulse oximetry and chest radiography, none of which are reliable for confirming tube position if used alone. The detection of carbon dioxide by an end-tidal carbon dioxide detector (ETCO_2_ detector) is a reliable approach to confirm the endotracheal tube placement in the operating room, emergency department, and pre-hospital setting, although it has some limitations (51,52)**.**

Procedures, transportation, and other movements in the emergency departments and ICUs might dislocate the endotracheal tube. Therefore, continuous monitoring of the endotracheal tube with capnography, particularly in the patients with adequately perfusion, can prohibit subsequent complications such as poor ventilation and oxygenation, which ultimately result in tracheal ischemia.

### Cricoid pressure

Cricoid pressure is used to prevent the regurgitation of gastric contents during anesthesia and facilitate the tracheal intubation with rapid sequence induction (53). When this technique is properly applied (with adequate training and experience), it is safe and effective. In the patients with a history of difficult intubation, it is more difficult to perform. The appropriate amount of pressure in this maneuver is between 30 and 40 mmHg. Under- or overestimation of the pressure would result in reflux and airway obstruction, respectively (54). Consequently, aspiration pneumonia and failed intubation may result and affect the patient’s condition by prolonging ICU stay, arterial hemodynamic, as well as airway injuries.

The results revealed that the designed and developed instrument acquired acceptable validity values. In addition, Chronbach’s alpha coefficient also showed an appropriate internal consistency in the domains.

## CONCLUSION

The developed questionnaire is a reliable and valid instrument for precisely determining the risk factors of PITS. This questionnaire can be used in epidemiologic studies in this field in the different countries.

## References

[1] Nouraei SR Battson RM Koury EF Sandhu GS Patel A. Adult post-intubation laryngotracheal stenosis: an underestimated complication of intensive care. J Intensive Care Soc 2009;10:229.

[2] Nouraei SA Ma E Patel A Howard DJ Sandhu GS. Estimating the population incidence of adult post-intubation laryngotracheal stenosis. Clin Otolaryngol 2007;32(5):411–2. 17883582 10.1111/j.1749-4486.2007.01484.x

[3] Grillo HC Donahue DM Mathisen DJ Wain JC Wright CD. Postintubation tracheal stenosis. Treatment and results. J Thorac Cardiovasc Surg. 1995;109(3):486–92; discussion 492–3. 7877309 10.1016/S0022-5223(95)70279-2

[4] Stauffer JL Olson DE Petty TL. Complications and consequences of endotracheal intubation and tracheotomy. A prospective study of 150 critically ill adult patients. Am J Med 1981;70(1):65–76. 7457492 10.1016/0002-9343(81)90413-7

[5] Grillo HC. Development of tracheal surgery: a historical review. Part 1: Techniques of tracheal surgery. Ann Thorac Surg 2003;75(2):610–9. 12607695 10.1016/s0003-4975(02)04108-5

[6] Mathias DB Wedley JR. The effects of cuffed endotracheal tubes on the tracheal wall. Br J Anaesth 1974;46(11):849–52. 4621305 10.1093/bja/46.11.849

[7] Ferdinande P Kim DO. Prevention of postintubation laryngotracheal stenosis. Acta Otorhinolaryngol Belg 1995;49(4):341–6. 8525831

[8] Mol DA De Villiers Gdu T Claassen AJ Joubert G. Use and care of an endotracheal/tracheostomy tube cuff—are intensive care unit staff adequately informed? S Afr J Surg 2004;42(1):14–6. 15181709

[9] Volpi D Lin PT Kuriloff DB Kimmelman CP. Risk factors for intubation injury of the larynx. Ann Otol Rhinol Laryngol 1987;96(6):684–6. 3688758 10.1177/000348948709600614

[10] Papla B Dyduch G Frasik W Olechnowicz H. Post-intubation tracheal stenosis—morphological-clinical investigations. Pol J Pathol 2003;54(4):261–6. 14998295

[11] Mark EJ Meng F Kradin RL Mathisen DJ Matsubara O. Idiopathic tracheal stenosis: a clinicopathologic study of 63 cases and comparison of the pathology with chondromalacia. Am J Surg Pathol 2008;32(8):1138–43. 18545144 10.1097/PAS.0b013e3181648d4a

[12] Grillo HC Mathisen DJ. Surgical management of tracheal strictures. Surg Clin North Am 1988;68(3):511–24. 3375956 10.1016/s0039-6109(16)44531-7

[13] Grillo HC Cooper JD Geffin B Pontoppidan H. A low-pressure cuff for tracheostomy tubes to minimize tracheal injury. A comparative clinical trial. J Thorac Cardiovasc Surg 1971;62(6):898–907. 4942973

[14] Zias N Chroneou A Tabba MK Gonzalez AV Gray AW Lamb CR Post tracheostomy and post intubation tracheal stenosis: report of 31 cases and review of the literature. BMC Pulm Med 2008;8:18. 18803874 10.1186/1471-2466-8-18 PMC2556644

[15] Zangi M Saadat S Nahidi S Svanström L Mohammadi R. Epidemiology of injuries in metropolitan Tehran, Iran: a household survey. Int J Inj Contr Saf Promot 2015;22(3):224–31. 24754492 10.1080/17457300.2014.908220

[16] Saghebi SR Zangi M Tajali T Farzanegan R Farsad SM Abbasidezfouli A The role of T-tubes in the management of airway stenosis. Eur J Cardiothorac Surg 2013;43(5):934–9. 22991458 10.1093/ejcts/ezs514

[17] Abbasidezfouli A Shadmehr MB Arab M Javaherzadeh M Pejhan S Daneshvar A Postintubation multisegmental tracheal stenosis: treatment and results. Ann Thorac Surg 2007;84(1):211–4. 17588414 10.1016/j.athoracsur.2007.03.050

[18] Shadmehr MB Farzanegan R Zangi M Mohammadzadeh A Sheikhy K Pejhan S Thyroid cancers with laryngotracheal invasion. Eur J Cardiothorac Surg 2012;41(3):635–40. 22219428 10.1093/ejcts/ezr131

[19] Farzanegan R Alijanipour P Akbarshahi H Abbasidezfouli A Pejhan S Daneshvar A Major airways trauma, management and long term results. Ann Thorac Cardiovasc Surg 2011;17(6):544–51. 21881347 10.5761/atcs.oa.11.01679

[20] Shadmehr MB Farzanegan R Graili P Javaherzadeh M Arab M Pejhan S Primary major airway tumors; management and results. Eur J Cardiothorac Surg 2011;39(5):749–54. 20943410 10.1016/j.ejcts.2010.08.047

[21] Abbasidezfouli A Akbarian E Shadmehr MB Arab M Javaherzadeh M Pejhan S The etiological factors of recurrence after tracheal resection and reconstruction in post-intubation stenosis. Interact Cardiovasc Thorac Surg 2009;9(3):446–9. 19531537 10.1510/icvts.2009.202978

[22] Moafian G Aghabeigi MR Heydari ST Hoseinzadeh A Lankarani KB Sarikhani Y. An epidemiologic survey of road traffic accidents in Iran: analysis of driver-related factors. Chin J Traumatol 2013;16(3):140–4. 23735547

[23] Esteller-Moré E Ibañez J Matiñó E Ademà JM Nolla M Quer IM. Prognostic factors in laryngotracheal injury following intubation and/or tracheotomy in ICU patients. Eur Arch Otorhinolaryngol 2005;262(11):880–3. 16258758 10.1007/s00405-005-0929-y

[24] Griesdale DE Bosma TL Kurth T Isac G Chittock DR. Complications of endotracheal intubation in the critically ill. Intensive Care Med 2008;34(10):1835–42. 18604519 10.1007/s00134-008-1205-6

[25] Schmidt UH Kumwilaisak K Bittner E George E Hess D. Effects of supervision by attending anesthesiologists on complications of emergency tracheal intubation. Anesthesiology 2008;109(6):973–7. 19034093 10.1097/ALN.0b013e31818ddb90

[26] Cooper JD Grillo HC. The evolution of tracheal injury due to ventilatory assistance through cuffed tubes: a pathologic study. Ann Surg 1969;169(3):334–48. 5266019 10.1097/00000658-196903000-00007 PMC1387433

[27] Koshkareva Y Gaughan JP Soliman AM. Risk factors for adult laryngotracheal stenosis: a review of 74 cases. Ann Otol Rhinol Laryngol 2007;116(3):206–10. 17419525 10.1177/000348940711600308

[28] Sangoseni O Hellman M Hill C. Development and Validation of a Questionnaire to Assess the Effect of Online Learning on Behaviors, Attitudes, and Clinical Practices of Physical Therapists in the United States Regarding Evidenced-based Clinical Practice. Internet Journal of Allied Health Sciences and Practice 2013;11(2):7.

[29] Polit DF Beck CT Owen SV. Is the CVI an acceptable indicator of content validity? Appraisal and recommendations. Res Nurs Health 2007;30(4):459–67. 17654487 10.1002/nur.20199

[30] Polit DF Beck CT. The content validity index: are you sure you know what’s being reported? Critique and recommendations. Res Nurs Health 2006;29(5):489–97. 16977646 10.1002/nur.20147

[31] Ayre C Scally AJ. Critical values for Lawshe’s content validity ratio: revisiting the original methods of calculation. Measurement and Evaluation in Counseling and Development 2014;47(1):79–86.

[32] Fleiss JL. Statical methods for rates and proportions. 2nd ed. John Wiley and Sons Inc: New York; 1981.

[33] Lynn MR. Determination and quantification of content validity. Nursing research 1986;35(6):382–6. 3640358

[34] Araban M Tavafian SS Motesaddi Zarandi S Hidarnia AR Gohari MR Prochaska JM Introducing a new measure for assessing self-efficacy in response to air pollution hazards for pregnant women. J Environ Health Sci Eng 2013;11(1):16. 24491221 10.1186/2052-336X-11-16 PMC3776291

[35] Stemler SE. A comparison of consensus, consistency, and measurement approaches to estimating interrater reliability. Practical Assessment, Research & Evaluation 2004;9(4):1–9.

[36] Grant JS Davis LL. Selection and use of content experts for instrument development. Res Nurs Health 1997;20(3):269–74. 9179180 10.1002/(sici)1098-240x(199706)20:3<269::aid-nur9>3.0.co;2-g

[37] Waltz CF Strickland OL Lenz ER, editors. Measurement in nursing and health research. Springer Publishing Company; 2010.

[38] Cicchetti DV Sparrow SA. Developing criteria for establishing interrater reliability of specific items: applications to assessment of adaptive behavior. Am J Ment Defic 1981;86(2):127–37. 7315877

[39] Sharyfy A Khatony A Rezaey M. Is there a relationship between core body temperature and changes of endotracheal tube cuff pressure?. Iran J Crit Care Nurs 2014;6(3):102–9.

[40] Nseir S Zerimech F Fournier C Lubret R Ramon P Durocher A Continuous control of tracheal cuff pressure and microaspiration of gastric contents in critically ill patients. Am J Respir Crit Care Med 2011;184(9):1041–7. 21836137 10.1164/rccm.201104-0630OC

[41] Lizy C Swinnen W Labeau S Poelaert J Vogelaers D Vandewoude K Cuff pressure of endotracheal tubes after changes in body position in critically ill patients treated with mechanical ventilation. Am J Crit Care 2014;23(1):e1–8. 24382623 10.4037/ajcc2014489

[42] Sole ML Aragon D Bennett M Johnson RL. Continuous measurement of endotracheal tube cuff pressure: how difficult can it be? AACN Adv Crit Care 2008;19(2):235–43. 18560292 10.1097/01.AACN.0000318126.79630.76 PMC3506175

[43] Atlas GM. A mathematical model of differential tracheal tube cuff pressure: effects of diffusion and temperature. J Clin Monit Comput 2005;19(6):415–25. 16437293 10.1007/s10877-005-1626-5

[44] Souza Neto EP Piriou V Durand PG George M Evans R Obadia JF Influence of temperature on tracheal tube cuff pressure during cardiac surgery. Acta Anaesthesiol Scand 1999;43(3):333–7. 10081541 10.1034/j.1399-6576.1999.430315.x

[45] Holzapfel L. Nasal vs oral intubation. Minerva Anestesiol 2003;69(5):348–52. 12768165

[46] Danzl DF Thomas DM. Nasotracheal intubations in the emergency department. Crit Care Med 1980;8(11):677–82. 7428396 10.1097/00003246-198011000-00019

[47] Cooper KL. Evidence-based prevention of pressure ulcers in the intensive care unit. Crit Care Nurse 2013;33(6):57–66. 10.4037/ccn2013985 24293556

[48] Campbell KE Woodbury MG Houghton PE. Implementation of best practice in the prevention of heel pressure ulcers in the acute orthopedic population. Int Wound J 2010;7(1):28–40. 20409248 10.1111/j.1742-481X.2009.00650.x PMC7951332

[49] Ozdemir H Karadag A. Prevention of pressure ulcers: a descriptive study in 3 intensive care units in Turkey. J Wound Ostomy Continence Nurs 2008;35(3):293–300. 18496086 10.1097/01.WON.0000319128.06607.ac

[50] Kim D Jeon B Son JS Lee JR Ko S Lim H. The changes of endotracheal tube cuff pressure by the position changes from supine to prone and the flexion and extension of head. Korean J Anesthesiol 2015;68(1):27–31. 25664152 10.4097/kjae.2015.68.1.27 PMC4318861

[51] Grmec S. Comparison of three different methods to confirm tracheal tube placement in emergency intubation. Intensive Care Med 2002;28(6):701–4. 12107674 10.1007/s00134-002-1290-x

[52] Rudraraju P Eisen LA. Confirmation of endotracheal tube position: a narrative review. J Intensive Care Med 2009;24(5):283–92. 19654121 10.1177/0885066609340501

[53] Landsman I. Cricoid pressure: indications and complications. Paediatr Anaesth 2004;14(1):43–7. 14717873 10.1046/j.1460-9592.2003.01202.x

[54] Brimacombe JR Berry AM. Cricoid pressure. Can J Anaesth 1997;44(4):414–25. 9104526 10.1007/BF03014464

[55] Fishman NH Dedo HH Hamilton WK Hinchcliffe WA Roe BB. Postintubation tracheal stenosis. Ann Thorac Surg 1969;8(1):47–56. 5785925 10.1016/s0003-4975(10)66407-7

[56] Miller DR Sethi G. Tracheal stenosis following prolonged cuffed intubation: cause and prevention. Ann Surg 1970;171(2):283–93. 5413464 10.1097/00000658-197002000-00018 PMC1396664

[57] Andrews MJ Pearson FG. Incidence and pathogenesis of tracheal injury following cuffed tube tracheostomy with assisted ventilation: analysis of a two-year prospective study. Ann Surg 1971;173(2):249–63. 5541484 10.1097/00000658-197102000-00012 PMC1397624

[58] Balluch H. Pathogenesis of tracheal stenosis following long-term intubation of patients with multiple injuries. HNO 1983;31(11):395–8. 6654713

[59] Kastanos N Estopá Miró R Marín Perez A Xaubet Mir A Agustí-Vidal A. Laryngotracheal injury due to endotracheal intubation: incidence, evolution, and predisposing factors. A prospective long-term study. Crit Care Med 1983;11(5):362–7. 6839788 10.1097/00003246-198305000-00009

[60] Whited RE. A prospective study of laryngotracheal sequelae in long-term intubation. Laryngoscope 1984;94(3):367–77. 6700353 10.1288/00005537-198403000-00014

[61] Bishop MJ. Mechanisms of laryngotracheal injury following prolonged tracheal intubation. Chest 1989;96(1):185–6. 2661158 10.1378/chest.96.1.185

[62] Grillo HC Donahue DM. Post intubation tracheal stenosis. Semin Thorac Cardiovasc Surg 1996;8(4):370–80. 8899924

[63] Vila J Bosque MD García M Palomar M Quesada P Ramis B. Endoscopic evolution of laryngeal injuries caused by translaryngeal intubation. Eur Arch Otorhinolaryngol 1997;254 Suppl 1:S97–100. 9065639 10.1007/BF02439735

[64] Beebe DS. Complications of tracheal intubation. InSeminars in Anesthesia, Perioperative Medicine and Pain 2001 9 1 (Vol. 20, No. 3, pp. 166–172). WB Saunders.

[65] Yamada Y Sugai M Woo M Nishida N Sugimoto T. Acquired subglottic stenosis caused by methicillin resistant Staphylococcus aureus that produce epidermal cell differentiation inhibitor. Arch Dis Child Fetal Neonatal Ed 2001;84(1):F38–9. 11124922 10.1136/fn.84.1.F38 PMC1721192

[66] Hocking G Roberts FL Thew ME. Airway obstruction with cricoid pressure and lateral tilt. Anaesthesia 2001;56(9):825–8. 11531665 10.1046/j.1365-2044.2001.02133.x

[67] Koufman JA Aviv JE Casiano RR Shaw GY. Laryngopharyngeal reflux: position statement of the committee on speech, voice, and swallowing disorders of the American Academy of Otolaryngology-Head and Neck Surgery. Otolaryngol Head Neck Surg 2002;127(1):32–5. 12161727 10.1067/mhn.2002.125760

[68] Zagalo C Santiago N Grande NR Martins dos Santos J Brito J Aguas AP. Morphology of trachea in benign human tracheal stenosis: a clinicopathological study of 20 patients undergoing surgery. Surg Radiol Anat 2002;24(3–4):160–8. 12375067 10.1007/s00276-002-0031-8

[69] Rangachari V Sundararajan I Sumathi V Kumar KK. Laryngeal sequelae following prolonged intubation: a prospective study. Indian Journal of Critical Care Medicine 2006;10(3):171.

[70] J Young P J Doyle A. Preventing ventilator-associated pneumonia-The role of the endotracheal tube. Current Respiratory Medicine Reviews 2012;8(3):170–83.

[71] Herrak L Ahid S Abouqal R Lescot B Gharbi N. Tracheal stenosis after intubation and/or tracheostomy. Egyptian Journal of Chest Diseases and Tuberculosis 2014;63(1):233–7.

[72] Hong SJ. Will the Taper Shaped Cuff Replace the Conventional High Volume-Low Pressured Cuff on Endotracheal Tube?. The Korean Journal of Critical Care Medicine 2014;29(1):1–2.

[73] Adigüzel N Karakurt Z Moçin ÖY Takır HB Saltürk C Kargın F Full-time ICU staff in the intensive care unit: Does it improve the outcome?. Turk Toraks Dergisi/Turkish Thoracic Journal 2015;16(1). 10.5152/ttd.2014.4317 PMC5783043 29404074

[74] Memela ME Gopalan PD. Variations in endotracheal tube cuff pressure: Is 8-hourly monitoring enough?. Southern African Journal of Critical Care 2014;30(2):35–40.

[75] Bauchmuller K Faulds MC. Care of the critically ill patient. Surgery (Oxford). 2015;33(4):165–71.

[76] Elmer J Lee S Rittenberger JC Dargin J Winger D Emlet L. Reintubation in critically ill patients: procedural complications and implications for care. Crit Care 2015;19:12. 25592172 10.1186/s13054-014-0730-7 PMC4328699

[77] Alzahrani AR Al Abbasi S Abahoussin OK Al Shehri TO Al-Dorzi HM Tamim HM Prevalence and predictors of out-of-range cuff pressure of endotracheal and tracheostomy tubes: a prospective cohort study in mechanically ventilated patients. BMC Anesthesiol 2015;15:147. 26471790 10.1186/s12871-015-0132-7 PMC4607016

